# Evolution of Pectobacterium Bacteriophage ΦM1 To Escape Two Bifunctional Type III Toxin-Antitoxin and Abortive Infection Systems through Mutations in a Single Viral Gene

**DOI:** 10.1128/AEM.03229-16

**Published:** 2017-03-31

**Authors:** Tim R. Blower, Ray Chai, Rita Przybilski, Shahzad Chindhy, Xinzhe Fang, Samuel E. Kidman, Hui Tan, Ben F. Luisi, Peter C. Fineran, George P. C. Salmond

**Affiliations:** a Department of Biochemistry, University of Cambridge, Cambridge, United Kingdom; b Department of Microbiology and Immunology, University of Otago, Dunedin, New Zealand; University of Tennessee and Oak Ridge National Laboratory

**Keywords:** type III toxin-antitoxin, ΦM1, Pectobacterium atrosepticum, abortive infection, bacteriophage-bacterium interaction

## Abstract

Some bacteria, when infected by their viral parasites (bacteriophages), undergo a suicidal response that also terminates productive viral replication (abortive infection [Abi]). This response can be viewed as an altruistic act protecting the uninfected bacterial clonal population. Abortive infection can occur through the action of type III protein-RNA toxin-antitoxin (TA) systems, such as ToxIN_Pa_ from the phytopathogen Pectobacterium atrosepticum. Rare spontaneous mutants evolved in the generalized transducing phage ΦM1, which escaped ToxIN_Pa_-mediated abortive infection in P. atrosepticum. ΦM1 is a member of the Podoviridae and a member of the “KMV-like” viruses, a subset of the T7 supergroup. Genomic sequencing of ΦM1 escape mutants revealed single-base changes which clustered in a single open reading frame. The “escape” gene product, M1-23, was highly toxic to the host bacterium when overexpressed, but mutations in M1-23 that enabled an escape phenotype caused M1-23 to be less toxic. M1-23 is encoded within the DNA metabolism modular section of the phage genome, and when it was overexpressed, it copurified with the host nucleotide excision repair protein UvrA. While the M1-23 protein interacted with UvrA in coimmunoprecipitation assays, a UvrA mutant strain still aborted ΦM1, suggesting that the interaction is not critical for the type III TA Abi activity. Additionally, ΦM1 escaped a heterologous type III TA system (TenpIN_Pl_) from Photorhabdus luminescens (reconstituted in P. atrosepticum) through mutations in the same protein, M1-23. The mechanistic action of M1-23 is currently unknown, but further analysis of this protein may provide insights into the mode of activation of both systems.

**IMPORTANCE** Bacteriophages, the viral predators of bacteria, are the most abundant biological entities and are important factors in driving bacterial evolution. In order to survive infection by these viruses, bacteria have evolved numerous antiphage mechanisms. Many of the studies involved in understanding these interactions have led to the discovery of biotechnological and gene-editing tools, most notably restriction enzymes and more recently the clustered regularly interspaced short palindromic repeats (CRISPR)-Cas systems. Abortive infection is another such antiphage mechanism that warrants further investigation. It is unique in that activation of the system leads to the premature death of the infected cells. As bacteria infected with the virus are destined to die, undergoing precocious suicide prevents the release of progeny phage and protects the rest of the bacterial population. This altruistic suicide can be caused by type III toxin-antitoxin systems, and understanding the activation mechanisms involved will provide deeper insight into the abortive infection process.

## INTRODUCTION

It is estimated that there are more than 10^30^ bacteriophages (phages) on Earth, outnumbering their bacterial hosts 10-fold (1, 2). These large viral numbers generate an estimated 10^25^ infections per second, imposing a large evolutionary selection pressure on bacteria (2). In response, bacteria have evolved a plethora of defensive mechanisms to counter these overwhelming phage insults (3). Consequently, phages are continually evolving counterdefenses, and thus both the host and parasite are locked together in a perpetual molecular arms race (4). Bacterial antiphage mechanisms that have been observed include adsorption prevention, restriction-modification systems, superinfection systems, abortive infection (Abi) systems, and the clustered regularly interspaced short palindromic repeats (CRISPR)-Cas systems (3). Studies of these phage-host interactions have been translated into significant molecular technologies and reagents, most notably the use of restriction enzymes in cloning (5) and, more recently, the CRISPR-Cas systems, the use of which is currently revolutionizing eukaryotic molecular biology (6).

One of the more curious antiphage mechanisms is Abi, in which, postinfection, the host bacterium is driven toward precocious cell death. This simultaneously terminates viral replication and prevents a productive phage burst. Thus, the Abi response in infected cells protects the bacterial population from progeny phage infection in a process akin to an altruistic suicide (3). The majority of Abi systems have been studied in Lactococcus lactis (7), an important bacterium in the dairy industry (8). Phage contamination in fermentation cultures can cause substantial economic losses. Consequently, considerable research has been conducted to identify and define many antiphage systems useful for control of bacteriophages in lactococcal fermentations (7). However, there are also well-studied Abi systems in other bacteria, such as Escherichia coli, namely, the Rex, Lit, and PrrC systems (9–11). A commonly recurring theme of Abi systems is that they involve the activation of a toxic protein that is suppressed under normal growth conditions. However, environmental insults, phages, or other physiological stresses can activate the toxin. Once activated, the toxin interferes with an essential cellular process and induces bacteriostasis, ultimately leading to cell death. This is a common feature shared by toxin-antitoxin (TA) systems (12).

TA systems were originally discovered on plasmids, where they function as plasmid maintenance systems through postsegregational killing mechanisms (13). They have been found in the majority of bacteria, both on plasmids (13) and chromosomally (14), as well as in archaea (15) and phages (16). TA systems are typically bicistronic, comprising a bacteriostatic or bactericidal toxic protein that is neutralized either directly or indirectly by an antitoxin counterpart. To date, there are six TA system types which are characterized by the nature and mode of action of their antitoxins (17). In the case of type III TA systems, an RNA antitoxin directly interacts with the toxic protein to form a nontoxic complex (18).

At least four types of TA systems confer phage resistance. These are the *hok/sok* systems of type I (19), *mazEF*, *rnlAB*, and *lsoAB* of type II (20, 21), ToxIN_Pa_, TenpIN_Pl_, and AbiQ of type III (22–24), AbiE of type IV (25), and sanaTA (which is currently not characterized but likely to be a type II, having a proteinaceous antitoxin) (26). ToxIN_Pa_ was the first type III system to be identified and originated from Pectobacterium atrosepticum plasmid pECA1039. The toxin ToxN_Pa_ is encoded by *toxN*, and the antitoxin ToxI_Pa_ is encoded by *toxI*, a 36-nucleotide sequence repeated five and a half times (22). The ToxIN_Pa_ system provides protection against multiple phages infecting not only its cognate host, P. atrosepticum, but also other enteric bacteria, including E. coli DH5α and Serratia marcescens Db11 (22). One such aborted pectobacterial phage is the Myoviridae phage ΦTE. ΦTE phages that were no longer sensitive to ToxIN_Pa_ had evolved to encode an RNA antitoxic mimic of ToxI_Pa_, which was able to neutralize ToxN_Pa_ (27). However, it did not shed light on how ToxIN_Pa_ was activated during phage infection. In fact, very little is known about the activation of any type III toxin-antitoxin systems. The other type III system that has been studied for Abi is AbiQ from Lactococcus lactis, which shows structural homology with ToxN_Pa_ (24). Three lactococcal siphophages that were aborted by AbiQ have been examined in detail. However, all had mutations in genes of unknown functions; *orf38*, *m1*, and *e19* of phages P008, bIL170, and c2, respectively (28). The AbiQ system was also reconstructed in a heterologous host, E. coli MG1655, and was shown to confer resistance to a range of coliphages, including T4 and T5. However, escape mutants could be obtained only for a single phage (phage 2). Escape mutants of this phage showed mutations in *orf210*, a predicted DNA polymerase (28). Studies of the AbiQ system suggests that there may be multiple potential routes of escape involving several genes from different phages in the activation of a single Abi system.

Previously it was shown that the pectobacterial phage ΦM1 was aborted by the ToxIN_Pa_ system and was able to escape by evolving rare mutants (29). ΦM1 was isolated in 1995 during a search for new transducing phages effective as genetic tools in P. atrosepticum (30). Here we characterize ΦM1 and its escape mutants in depth. All ΦM1 escape phages evolved through mutations in a gene encoding a small, highly toxic protein, M1-23. When the related TenpIN_Pl_ system of Photorhabdus luminescens was transferred to P. atrosepticum, the system was able to abort ΦM1 in the heterologous host. Furthermore, it was possible to select spontaneous viral mutants that escaped both ToxIN_Pa_ and TenpIN_Pl_ through mutations in M1-23.

## RESULTS

### ΦM1 is a “KMV-like” virus.

ΦM1 is a generalized transducing phage of Pectobacterium atrosepticum (previously Erwinia carotovora subsp. atroseptica) (30). This podovirus is aborted by the type III TA system, ToxIN, from P. atrosepticum, namely, ToxIN_Pa_ (22). ΦM1 generates spontaneous escape mutants that are resistant to Abi by ToxIN_Pa_ at a rate of ∼10^−5^ (29). In order to improve our understanding of ToxIN_Pa_-phage interactions, we sequenced ΦM1 wild type (wt) and three previously isolated escape phages, ΦM1-A, -B, and -D (29).

Using BLAST searches (31), ΦM1 was classified as a member of the “KMV-like” subgroup of the T7 supergroup of phages (32). T7-like phage linear genomes are typically flanked by direct terminal repeats (DTRs) (33). However, the DTRs could not be defined by a primer walking strategy along the ΦM1 genome, consistent with results from another KMV-like phage, LIMEzero (34). The presence and approximate size of the DTRs, 293 bp, were therefore confirmed through restriction digest analysis of the ΦM1 genome (see Fig. S2 in the supplemental material). The final ΦM1 wild-type genome was 43,827 bp long with a GC content of 49.30%. In comparison, the host P. atrosepticum genome has a GC content of 50.97% (35). The two genomes therefore closely match each other in GC content.

Global nucleotide alignments were performed to assess the relationship between the KMV-like phages and ΦM1. Compared with ΦM1, phage VP93 (43,931 bp) (36), phage LKA1 (41,593 bp) (32), phage LKD16 (43,200 bp) (32), and ΦKMV itself (42,519 bp) (33) shared between 48.2% and 49.2% sequence identity. These values match well those of other KMV-like phages (34).

**FIG 1:**
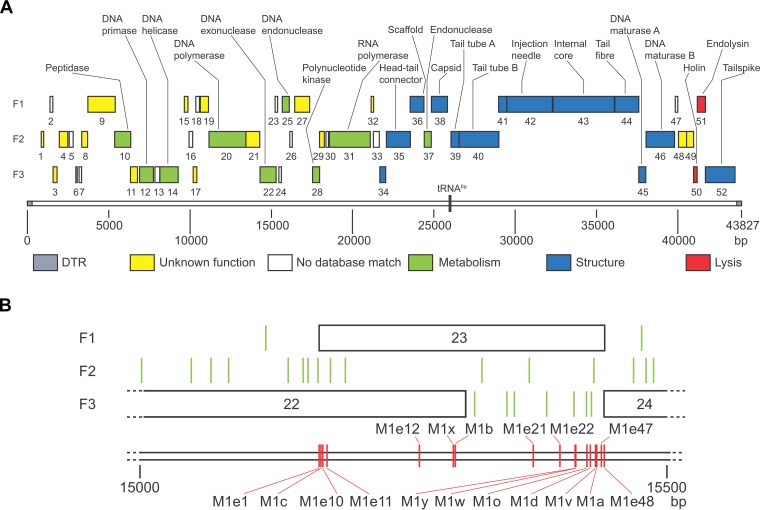
Genomic map of ΦM1 wild type and its escape locus. (A) All 52 annotated ORFs are coded on the forward reading strand, in a linear progression from metabolic genes to structural genes and, finally, to host cell lysis genes. Each forward reading frame is labeled F1, F2, or F3. ORFs are shown to scale as shaded boxes numbered with the gene number, colored according to the predicted role. The single tRNA^Ile^ gene is positioned on the scale, shown in purple. Where it was possible to identify a protein by homology searches, that ORF is labeled. The scale is in base pairs. The figure was drawn to scale using Adobe Illustrator. (B) Schematic of the escape locus of ΦM1. All escape phage mutations are within *phiM1-23*. Each forward reading frame is labeled F1, F2, or F3. Each ORF is shown to scale as a box, numbered with the gene number. Each stop codon is represented as a green vertical line. The positions of the ΦM1 escape phage mutations are shown by red vertical lines, labeled with the parent phage. The scale is in base pairs.

ΦM1 contains 52 putative genes, named *phiM1-1* to *phiM1-52*. The gene products were named M1-1 to M1-52, and they are encoded by 92.6% of the genome. Subsequent BLASTp searches identified homologues for 32 of the open reading frames (ORFs) from other KMV-like phages (Table S1). In most cases, it was therefore possible to assign putative functions and categorize ORFs as containing either metabolism, structural, or host lysis genes (Fig. 1A). ΦM1 also encodes a single tRNA^Ile^, between *phiM1-38* and *phiM1-39*.

### ΦM1 escape mutations had specific base substitutions.

The genome sequences of the three escape phages, M1-A, -B, and -D, were compared with that of the wt. All three escape phages had single point mutations localized to a 124-bp stretch (Fig. 1B), across *phiM1-22* and *phiM1-23*, which we refer to as the “escape locus.” To ascertain whether these point substitutions were individual changes, further escape phages were isolated using independent lysates to avoid the possibility of sibling mutants. The new escape phage mutants were isolated following selection on P. atrosepticum pTA46 (ToxIN_Pa_) (22, 29). The escape locus of each phage was sequenced following amplification of the region from the purified genomic DNA. We observed that all 10 escape phages had unique mutations distributed across 246 bp of the escape locus (Fig. 1B). Nine of these mutations were base substitutions, while one was a single base deletion (Table 1).

**TABLE 1:** Summary of ΦM1 escape mutations and effects on reading frames

Phage	Date of isolation	Position and mutation relative to ΦM1 wt^*a*^	Effect on forward reading frame^*b*^:		
			F1	F2	F3
ΦM1-A	March 2007	15416, A to C	Y to S	T to P	No change
ΦM1-B	March 2007	15292, C to T	R to stop	No change	P to L
ΦM1-C	March 2007	15170, T to C	M to T	Stop to S	No change
ΦM1-D	March 2007	15410, T to C	M to T	W to R	No change
ΦM1-O	June 2009	15407, A to C	Q to P	No change	No change
ΦM1-V	May 2009	15415, T to G	Y to D	No change	V to G
ΦM1-W	May 2009	15398, A to T	D to V	M to L	Stop to C
ΦM1-X	May 2009	15288, AA to A	FS to stop after 9 aa (wild-type F1 continues *phiM1-23*)	FS causing Q to H and stop after 3 aa (wild-type F2 stops after 9 aa)	FS causing N to T and shift of ORF1 into ORF2 (wild-type F3 stops after 3 aa)
ΦM1-Y	May 2009	15397, G to A		
ΦM1-Z	May 2009	15416, A to G (cf. ΦM1-A)	

a Mutations are indicated as, e.g., Y to S.

b FS, frameshift; aa, amino acid.

### Infection with ΦM1 affects the ToxI_Pa_/ToxN_Pa_ ratio.

**FIG 2:**
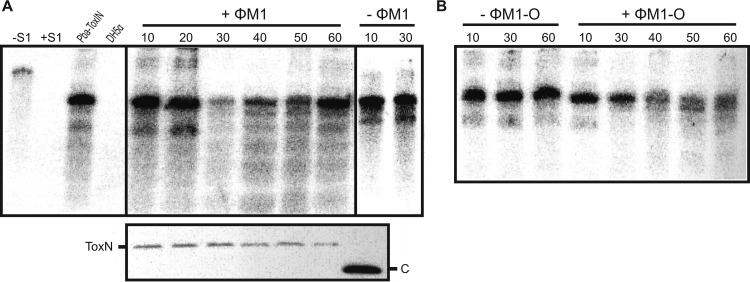
ToxI_Pa_ levels are affected during phage infection. (A) S1 nuclease assay targeting the full 5.5-repeat ToxI_Pa_ sequence was used to monitor ToxI_Pa_ levels during ΦM1 infection. Assays were performed on 10 μg total RNA prepared from P. atrosepticum ToxIN_Pa_ (pMJ4) at different times following ΦM1 infection. Numbers indicate the time (minutes) after infection with phage (+ΦM1) and the negative control without phage (−ΦM1). Hybridization to total RNA from P. atrosepticum expressing ToxIN_Pa_ (pTA46) and DH5α served as positive and negative controls, respectively. The expression of ToxN_Pa_ at the respective time points of infection is shown in the lower panel using Western blotting; “C” indicates the 11-kDa SdhE-FLAG protein used as a loading and size control (54). (B) S1 nuclease assay targeting ToxI_Pa_ for the infection with the escape phage ΦM1-O. The assay was done as described for panel A.

Though it has been shown that ToxN_Pa_ levels do not alter during a ΦM1 phage infection (29), it was not known how the ToxI_Pa_ levels were affected. The identification of the escape phages provided an opportunity to address this question. To investigate alterations to the ToxI_Pa_/ToxN_Pa_ ratio, we monitored the levels of ToxI_Pa_ and ToxN_Pa_-FLAG during the infections by ΦM1 and the escape phage ΦM1-O within P. atrosepticum carrying a ToxIN_Pa_-FLAG plasmid (pMJ4). Total protein and RNA samples were taken at different times after infection and subjected to Western blotting and an S1 nuclease assay, respectively. While ToxN_Pa_ levels stayed constant throughout infection (Fig. 2A, lower panel), ToxI_Pa_ levels dropped dramatically after 30 min compared to those of an uninfected control (Fig. 2A). Interestingly, ToxI_Pa_ levels increased back to original levels at 60 min. In comparison to the infection with ΦM1 wt, ToxI_Pa_ levels did not change significantly at 30 min during infection with the escape phage ΦM1-O (Fig. 2B). The ToxI_Pa_ level did decrease with the ΦM1-O infection but only at 40 min (Fig. 2B). The ToxI_Pa_ levels were not then restored, as in the case of ΦM1 wt (Fig. 2B). ΦM1 appears to activate ToxN_Pa_, and thereby initiate Abi, by causing a decrease in the cellular ToxI_Pa_ levels, either through direct or indirect means. In the case of ΦM1-O, this activation is prevented due to the mutation in M1-23. This would allow the phage to propagate, which may then account for the delayed decrease and lack of restoration in ToxI_Pa_ levels.

### Identification and characterization of the ΦM1 escape product.

The majority of escape mutations occurred within *phiM1-23*. On first analysis, two mutations, those from ΦM1-B and ΦM1-X, occurred at the 3′ end of *phiM1-22*. Another mutation, from ΦM1-C, mapped further upstream, again within *phiM1-22*. This gene, *phiM1-22*, encodes a homologue of a putative DNA exonuclease from phage LKA1 (Table S1) (32). Unfortunately, there were no database hits for *phiM1-23* and *phiM1-24*, using either the nucleotide or encoded protein sequences.

**FIG 3:**
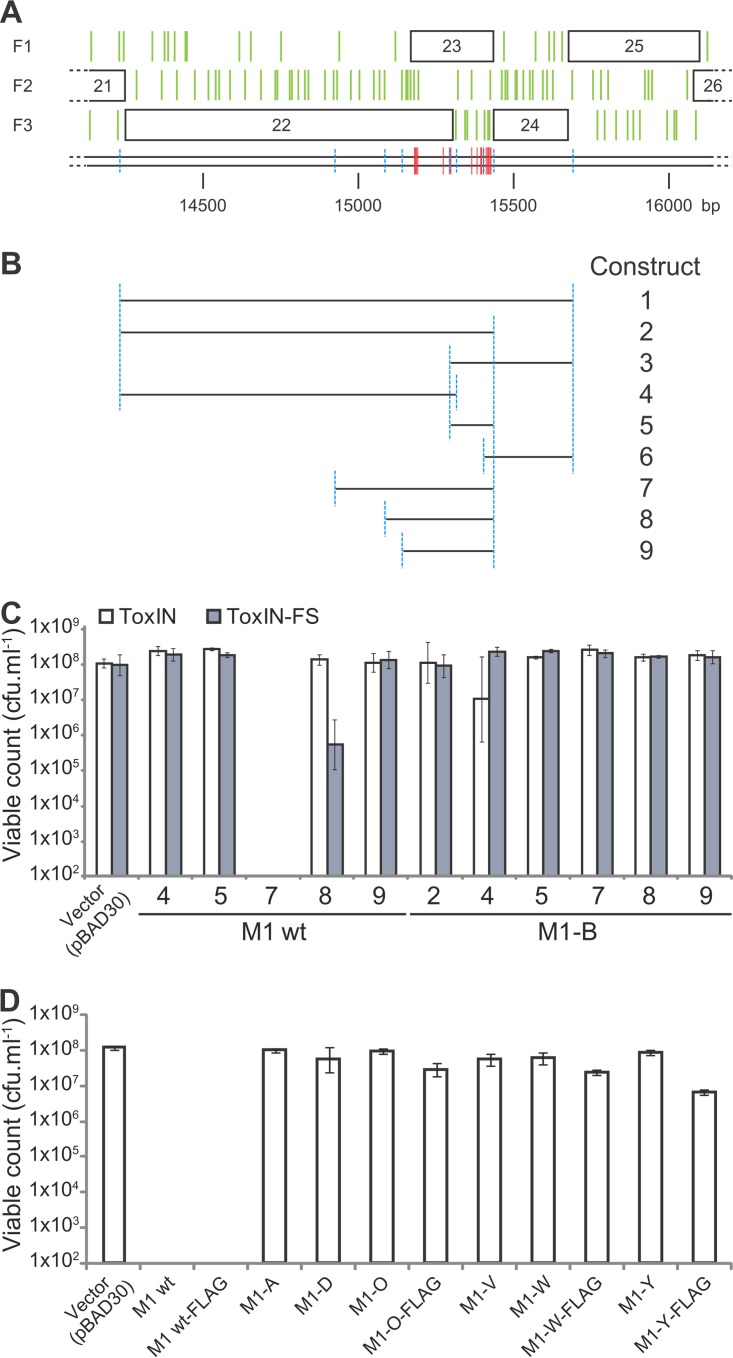
Toxicity of the ΦM1 escape locus products. (A) The escape locus of ΦM1 as described in the legend to Fig. 1B. The positions of the ΦM1 escape phage mutations are shown by red vertical lines, labeled with the parent phage. The scale is in base pairs. (B) Specific regions of the phage genomes, designated by the length of the line that corresponds to the genomic locus shown in panel A, were cloned into pBAD30 to make nine different constructs. Blue dashed lines in panel A reflect the construct boundaries in panel B. The figure is drawn to scale. (C) Expression of ΦM1 wt and ΦM1-B escape loci in P. atrosepticum. Strains of P. atrosepticum containing either a ToxIN_Pa_ or ToxIN_Pa_-FS plasmid (pTRB125 or pTRB126), together with a phage construct (or pBAD30 vector control), were tested for toxicity. (D) A range of construct 7 plasmids was tested for toxicity in P. atrosepticum. The escape phage constructs were all reduced for toxicity. Error bars show the standard deviations for triplicate data.

Specific regions of this escape locus were amplified from ΦM1 phages and then cloned into pBAD30 (37) to make inducible constructs (Fig. 3A and B). The cloning began with constructs 1 to 6, using DNA from ΦM1 wt and ΦM1-B (Fig. 3B). Constructs 1 and 2 could not be obtained with ΦM1 wt DNA, presumably through toxicity of the resulting wt constructs in E. coli DH5α, but could be made using ΦM1-B escape phage DNA. Constructs 3, 4, 5, and 6 could be made using both sources of DNA. Due to the regions covered by these constructs, we could determine that within this locus, the genes of interest were *phiM1-22* and *phiM1-23* and that *phiM1-24* did not contribute to toxicity. As pBAD30 is tightly repressed by glucose in E. coli DH5α, this also implied that toxicity from this region of DNA might be occurring via an internal promoter.

Upon first analysis, the putative ATG start of *phiM1-23* was at bp 15304. Taking into account the stop codons of each frame (Fig. 1B, green vertical lines), the putative ATG start codon of *phiM1-23* could theoretically have been upstream of this initial annotation. There were three possible ATG sites upstream of the putative start codon for *phiM1-23*. The mutation of ΦM1-C specifically altered the middle of these start codons from M to T (Table 1). This start codon also had a ribosome binding site closer to consensus than those of the other potential start codons, making it the most obvious candidate. If this were the case, the escape mutations would span *phiM1-23* specifically. Constructs 7 to 9 were designed and made in order to test whether *phiM1-23* alone could generate a toxic phenotype.

We performed experiments to assess the toxicity of the escape locus constructs and to determine whether toxicity was related to the presence of ToxIN_Pa_. P. atrosepticum was transformed with inducible derivatives of the escape locus in combination with either pBluescript-based (Fermentas) ToxIN_Pa_ or negative-control ToxIN_Pa_-frameshift (FS) vectors (pTRB125 and pTRB126, respectively). Serial dilutions of these dual-vector strains of P. atrosepticum were then incubated with and without induction, overnight, to determine the viable count (Fig. 3C). This clearly showed that the product of construct 7, covering *phiM1-23* specifically, was toxic. There was no toxicity in the case of ΦM1-B, the mutation in which causes a premature stop codon in *phiM1-23*. Toxicity was also independent of the presence of ToxIN_Pa_. These results strongly suggested that *phiM1-23* produces a small, toxic protein, responsible either directly or indirectly for activation of Abi against ΦM1.

New versions of construct 7 (Fig. 3D) were then generated, with the addition of a C-terminal FLAG tag to the M1-23 product, using both ΦM1 wt and escape sequences. Various constructs were then tested for toxicity in the cognate host, P. atrosepticum (Fig. 3D). All the escape constructs tested showed reduced toxicity (Fig. 3D). It was therefore possible to attempt overexpression and purification of M1-23, using an E. coli expression strain, ER2566. After expression trials using constructs made from ΦM1 wt and ΦM1-O, -W, and -Y phage DNA, the M1-O-23FLAG product was chosen for further study. Sufficient M1-O-23FLAG protein was purified to allow mass spectrometry to confirm both the identity of the protein and, specifically, the presence of the expected Q-to-P mutation. Furthermore, the protein sample was subjected to N-terminal sequencing, generating a sequence of TKM. This implied that *phiM1-23* started at the ATG specifically mutated by ΦM1-C, as described earlier, and that the initial methionine is cleaved posttranslationally. The annotation of the ΦM1 wt genome was then altered to accommodate *phiM1-23* beginning at this confirmed start codon. In summary, this result shows that all the escape mutations map to a single gene, *phiM1-23*, which generates a 9.8-kDa protein. These mutations reduce the toxicity of the protein product and allow viral escape from ToxIN_Pa_-induced Abi.

It had not been possible to clone constructs 1 and 2 (Fig. 3B) using the ΦM1 wt sequence, despite the pBAD30 vector system being repressed in the presence of glucose. This suggested that a promoter internal to those cloned regions might be inducing the transcription of *phiM1-23*. A range of pRW50-based (38) *lacZ* transcriptional fusion constructs was generated to investigate the possible presence of a promoter (Fig. S3A). In this case, it was possible to clone the equivalent of construct 2 using ΦM1 wt DNA (Fig. 3B), perhaps due to pRW50 having a low copy number, so the level of toxicity was sufficiently low. Plasmid pTA104 (22), containing the promoter for ToxIN_Pa_, was used as a positive control. All the test constructs except pTRB162, which was an extremely truncated clone, generated LacZ activity (Fig. S3B). This confirmed the presence of a weak *phiM1-23* promoter within *phiM1-22*.

### Extensive analysis of ΦM1 escape mutants map all mutations to *phiM1-23*.

The initial 10 escape mutants of ΦM1 all had unique mutations in M1-23, so it was likely that there were other possible mutations not yet observed. Identifying these other mutations could reveal important residues involved in the functionality of M1-23. Consequently, a larger library of escape mutants was isolated and characterized in the same way as the initial escape mutants. A total of 51 new, independent escape phages were isolated, and their *phiM1-23* sequences were characterized. All escape phages were shown to have a mutation in this region, and several new unique escape phages were isolated (Table S2). With the addition of these new escape phages, the number of different mutations increased to 20. Interestingly, mutations in all three of the bases of the putative start codon were isolated, consistent with this being the correctly annotated start site. Other interesting mutations were those causing N-terminally located truncations of M1-23. In particular, ΦM1-E11 produced only a hypothetical dipeptide or indeed just a single amino acid if the initial starting methionine was removed. Although most mutations in M1-23 were missense alleles generating single amino acid residue changes, the ability to isolate derivatives with major truncations showed that the M1-23 protein must be nonessential for a productive ΦM1 lytic cycle. Other notable mutations were ΦM1-E48 and ΦM1-E49 (both generating the same outcome), which modify the stop codon and lead to a 10-amino-acid C-terminal extension. It is puzzling why the 10-mer extension might impact function, because the addition of the octameric FLAG tag to the C terminus of M1-23 did not disrupt protein toxicity. Perhaps the extension might harbor a sequence that could act as an autoinhibitor or disrupt protein structure.

### M1-23 interacts with UvrA, but abortive infection can still take place in UvrA-deficient P. atrosepticum.

To assess whether there is a direct interaction of M1-23 with the ToxIN_Pa_ complex, His-tagged forms of both M1-23 and M1-O-23 were cloned, allowing overexpression and purification of these proteins. Coimmunoprecipitation reactions were carried out, but the results showed no evidence for interactions between M1-23 and the ToxIN_Pa_ complex and no impact of M1-23 on the ToxI RNA (data not shown).

**FIG 4:**
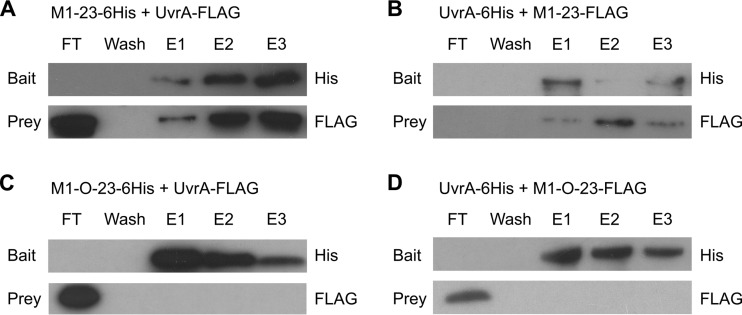
Coimmunoprecipitation of M1-23, M1-O-23, and UvrA. (A and B) Coimmunoprecipitation experiments with wild-type M1-23 and UvrA. (A) M1-23-6His was used as the bait and attached to a Ni^+^ column with UvrA-FLAG passed through. (B) The reciprocal experiment was performed with UvrA-6His used as the bait with M1-23-FLAG passed through. (C and D) The same coimmunoprecipitation experiments as described for panels A and B but using M1-O-23 instead of M1-23. (C) M1-O-23 was used as bait; (D) UvrA-6His was used as bait.

During the process of purifying M1-23-6His, it was noted that an additional high-molecular-weight band that was not present in control samples appeared in the eluted sample, and it was then copurified with M1-23 following ion-exchange fast performance liquid chromatography (FPLC; data not shown). Mass spectrometric analysis identified the host nucleotide excision repair protein, UvrA. Reciprocal coimmunoprecipitation assays were performed using purified protein samples to confirm this interaction (Fig. 4). M1-23 protein retained UvrA, while M1-O-23 did not, and similarly, only M1-23 was retained by immobilized UvrA (Fig. 4). This strongly suggests that M1-23 is a viral product that is able to bind host UvrA.

To assess potential effects of UvrA on abortive infection, a *uvrA* mutant was constructed in P. atrosepticum and confirmed by sequencing and then by hypersensitivity to UV light (Fig. S1). This strain was tested for its ability to abort ΦM1 via the ToxIN_Pa_ system. Surprisingly, ΦM1 was still aborted in the *uvrA* mutant and to the same extent as in the wild-type P. atrosepticum strain (efficiency of plating [EOP] of ΦM1 on the *uvrA* mutant with ToxIN_Pa_, 1.1 × 10^−5^). Escape phages of ΦM1 were isolated from the *uvrA* mutant, and their DNA was sequenced. Interestingly, all escape phages isolated on the *uvrA* mutant, ΦM1-U1, -U2, and ΦM1-U4 to ΦM1-U10 (which were independently isolated), carried mutations in the M1-23 sequence (Table S2). The results suggest that although M1-23 clearly has a specific interaction with UvrA, it appears that the escape route is either subtle or occurs indirectly.

### The ΦM1 escape mechanism works in another type III TA and Abi system.

Two further families of type III TA systems were recently identified, CptIN and TenpIN (23). TenpIN_Pl_, from the chromosome of Photorhabdus luminescens TT01, was able to act as an Abi system against coliphages when cloned on a multicopy plasmid and tested in an E. coli background (23). By transforming P. atrosepticum SCRI1043 with the TenpIN_Pl_ expression plasmid, pFR2 (23), we were able to test three Pectobacterium phages against the Abi activity of TenpIN_Pl_ (Table 2). While ΦS61 (29) and ΦTE (27) were dramatically affected by ToxIN_Pa_, neither were inhibited by TenpIN_Pl_ (Table 2). This indicates a degree of selectivity between the two Abi systems. ΦM1, however, was aborted by both systems, though to different degrees, which also underlines the selectivity under which ToxIN_Pa_ and TenpIN_Pl_ appear to operate. As with ToxIN_Pa_, it was possible to select for phages of ΦM1 that escaped Abi by TenpIN_Pl_. One of these escape phages, ΦM1-PL2, was isolated and sequenced. This escape phage had a single base substitution, T15410C, the same mutation as in ΦM1-D. To test this in reverse, escape phage ΦM1-O, selected with ToxIN_Pa_, was tested against TenpIN_Pl_ (Table 2). ΦM1-O was also resistant to TenpIN_Pl_. These results imply that in the case of ΦM1, the two systems operate in a similar fashion with a single protein, M1-23, being a key mediator.

**TABLE 2:** EOPs against ToxIN_Pa_ and TenpIN_Pl_ type III TA systems

Phage	EOP vs ToxIN_Pa_	EOP vs TenpIN_Pl_	System on which escape was selected
ΦM1 wt	1.3 × 10^−5^	1.1 × 10^−2^	
ΦS61	<3.2 × 10^−9^	0.9	
ΦTE	1.0 × 10^−8^	0.7	
ΦM1-O	1.0	1.0	ToxIN_Pa_
ΦM1-PL2	0.9	0.9	TenpIN_Pl_

## DISCUSSION

The pectobacterial phage ΦM1 was shown previously to be sensitive to the ToxIN_Pa_ system and capable of producing spontaneous escape mutants (29). Here we found that the ΦM1 phage is also sensitive to TenpIN_Pl_ when reconstructed in P. atrosepticum and is correspondingly able to evolve escape mutants. This is the first time we have been able to identify a phage that is able to escape the TenpIN_Pl_ system, and so further study may provide information about its activation. Interestingly, the ΦM1 phage is insensitive to two other type III systems tested, ToxIN_Bt_ from Bacillus thuringiensis and the CptIN_Er_ system from Eubacterium rectale (data not shown), and no Abi activity has so far been observed in these two systems (23, 39). In contrast, the P. atrosepticum phage ΦTE is aborted by ToxIN_Pa_ and able to escape the system by RNA-based molecular mimicry of the antitoxin (27) but is not aborted by the TenpIN_Pl_ system (Table 2).

Characterization of the ΦM1 phage in this study has shown that all escape mutants selected on ToxIN_Pa_ or TenpIN_Pl_ have mutations in a gene encoding M1-23. Alteration of single amino acids, extreme truncations due to very 5′ stop codons, or even stop codon mutations leading to short C-terminal extensions of M1-23 cause insensitivity to both ToxIN_Pa_ and TenpIN_Pl_. Escape mutants selected on one system are also insensitive to the other system, suggesting that there is a common pathway for the ΦM1 phage in the activation of these two systems. The role of M1-23 is unknown, but it was shown to be nonessential, and as it is located between a predicted exonuclease gene, *phiM1-22*, and a predicted endonuclease gene, *phiM1-25*, it might have a role in the regulation of nucleases or indeed may be able to act as a nuclease itself. In a previous study, it was shown that ToxN_Pa_ levels do not change during infection of the ΦM1 phage (29). In this study, we found that the ToxI_Pa_ levels decrease 30 min postinfection. In contrast, during the infection by the ΦM1 escape phage ΦM1-O, ToxI_Pa_ levels decreased only slightly after 40 min and were not restored. It appears that wild-type ΦM1 activates ToxN_Pa_ by decreasing the levels of ToxI_Pa_ and therefore initiating Abi. For ΦM1-O, the mutation in M1-23 prevents this early activation and thereby provides a window of opportunity for the phage to replicate.

To investigate the mechanism of M1-23 action, a large number of ΦM1 escape phages were isolated and their *phiM1-23* regions were sequenced. The results showed a number of escape mutations near the 5′ end of the gene, resulting in extremely truncated versions of the protein. This confirms that M1-23 is a nonessential viral protein. However, the majority of mutations found were toward the 3′ region of the gene and were mostly missense mutations resulting in single amino acid changes, implying that the C-terminal domain of the protein is important for Abi functionality. To further characterize M1-23, it was overexpressed and purified, but due to high toxicity, only a small amount of protein could be produced. Using the limited amount of protein available, interaction studies were performed to see if M1-23 interacted with ToxIN_Pa_. During purification of M1-23, a high-molecular-weight protein always copurified. Mass spectrometry of this protein confirmed that it was the DNA repair protein UvrA. It was shown through coimmunoprecipitation experiments that while M1-23 could interact with UvrA, the escape version of the protein M1-O-23 could not.

UvrA forms part of the SOS response in bacteria, a DNA damage response pathway (40) that has previously been shown to be involved in TA activation. The type I TA system TisB-IstR is under direct SOS response control, as *tisAB*, which encodes the TisB toxin, contains a LexA operator region that is inhibited by LexA (41). In addition to the SOS response, the stringent response has also been shown to play a role in the activation of TA systems. Both type I and type II TA systems have been shown to be regulated by (p)ppGpp, the central regulator of the stringent response (42, 43). However, ΦM1 and ΦTE were tested in a (p)ppGpp-negative double mutant (*relA spoT*) and were still aborted in that background (data not shown).

During the course of this study, the genomes of two new pectobacterial phages were sequenced. These were P. atrosepticum phage Peat1 (44) (GenBank accession number KR604693) and P. carotovorum phage PPWS1 (45) (DDBJ accession number LC063634). Both of these were podoviruses that shared high sequence identity to ΦM1. Peat1 (45,633 bp) shared 77.7% sequence identity, and PPWS1 (44,539 bp) shared 59.7% sequence identity. Furthermore, analysis of the two genomes revealed that both phages encoded M1-23 homologs, with the Peat1 homolog differing by only a single amino acid. Therefore, it is highly likely that both phages would be aborted by both the ToxIN_Pa_ and TenpIN_Pl_ systems and evolve escapes in the same way. If this was the case, it would show a common route through which phages of different bacteria are able to escape the same system.

Both ToxIN_Pa_ and TenpIN_Pl_ are very powerful antiphage abortive infection systems that belong to two different families of type III TA systems and are effective against a wide variety of phages. While many phages show differing sensitivities to the two systems, this study has shown that in ΦM1, there is a common pathway through which these two families of type III TA systems can be activated. This pathway involves a small toxic protein, M1-23, of unknown metabolic function that does not directly interact with the ToxIN_Pa_ complex but that interacts directly with UvrA. ΦM1 infection causes a diminution in ToxI_Pa_ levels, presumably leading to the destabilization of the ToxIN_Pa_ complex and consequent liberation of ToxN_Pa_ to induce cell death and concomitant abortive infection of the viral parasite.

## MATERIALS AND METHODS

### Bacterial strains, bacteriophages, and growth conditions.

Bacterial strains and bacteriophages are listed in Table 3. E. coli strains were grown at 37°C, and Pectobacterium atrosepticum SCRI1043 (35) was grown either at 25°C on agar plates or at 25, 28, or 30°C as required for liquid culture in Luria broth (LB) at 250 rpm or on LB agar (LBA). LBA contained 1.5% (wt/vol) or 0.35% (wt/vol) agar, to make LBA plates or top-LBA, respectively. Bacterial growth was measured using a spectrophotometer set to 600 nm. When required, media were supplemented with ampicillin (Ap) at 100 μg ml^−1^, chloramphenicol (Cm) at 50 μg ml^−1^, kanamycin (Km) at 50 μg ml^−1^, tetracycline (Tc) at 10 μg ml^−1^, isopropyl β-d-thiogalactopyranoside (IPTG) at 0.5 mM, or 2, 6-diaminopimelic acid (DAPA) at 300 μM. Spontaneous escape phage mutants were isolated as described previously (27). Phage lysates were made as described previously (46). Phages were stored at 4°C in phage buffer, i.e., 10 mM Tris-HCl (pH 7.4), 10 mM MgSO_4_, and 0.01% ^(^wt/vol) gelatin. A few drops of chloroform saturated with sodium bicarbonate was also added to the phage lysates to maintain sterility. EOP was calculated after overnight incubation of serial dilutions of phage lysates in a top-LBA lawn of each bacterial host and recorded as the number of PFU on the test strain relative to the number of PFU on the control strain. EOPs were calculated using P. atrosepticum wt or a frame-shifted *toxN* plasmid strain as the negative control (22).

**TABLE 3:** Bacterial strains and bacteriophages used in this study

Bacterium or phage	Genotype or characteristics	Reference or source
Bacteria		
Escherichia coli β2163	F^−^ RP4-2-Tc::Mu *dapA*::(*erm-pir*) Km^r^	57
E. coli DH5α	F^−^ *endA1 glnV44 thi-1 recA1 relA1 gyrA96 deoR nupG purB20* ϕ80d*lacZ*ΔM15 Δ(*lacZYA-argF*)*U169 hsdR17*(r_K_^−^ m_K_^+^) λ^−^	Gibco/BRL
E. coli ER2566	F^−^ λ^−^ *fhuA2* [*lon*] *ompT lacZ*::T7 gene 1 *gal sulA11* Δ(*mcrC-mrr*)114::IS*10* R(*mcr-73*::miniTn*10*-TetS)2 R(*zgb-210*::Tn*10*) (TetS) *endA1* [*dcm*]	NEB
E. coli W3100	F^−^ λ^−^ *rph-1* INV(*rrhD*, *rrhE*)	58
Pectobacterium atrosepticum SCRI1043	Wild-type strain	35
Phages		
ΦM1	Podoviridae, propagated on wt SCRI1043	30
ΦM1-A	ToxIN_Pa_ escape mutant of ΦM1	29
ΦM1-B	ToxIN_Pa_ escape mutant of ΦM1	29
ΦM1-C	ToxIN_Pa_ escape mutant of ΦM1	29
ΦM1-D	ToxIN_Pa_ escape mutant of ΦM1	29
ΦM1-O	ToxIN_Pa_ escape mutant of ΦM1	This study
ΦM1-V	ToxIN_Pa_ escape mutant of ΦM1	This study
ΦM1-W	ToxIN_Pa_ escape mutant of ΦM1	This study
ΦM1-X	ToxIN_Pa_ escape mutant of ΦM1	This study
ΦM1-Y	ToxIN_Pa_ escape mutant of ΦM1	This study
ΦM1-Z	ToxIN_Pa_ escape mutant of ΦM1	This study
ΦM1-Q	ToxIN_Pa_ escape mutant of ΦM1	This study
ΦM1-E1 to -E49	ToxIN_Pa_ escape mutant of ΦM1	This study
ΦM1-U1	ToxIN_Pa_ escape mutant of ΦM1 on UvrA mutant	This study
ΦM1-U2	ToxIN_Pa_ escape mutant of ΦM1 on UvrA mutant	This study
ΦM1-U4	ToxIN_Pa_ escape mutant of ΦM1 on UvrA mutant	This study
ΦM1-U5	ToxIN_Pa_ escape mutant of ΦM1 on UvrA mutant	This study
ΦM1-U6	ToxIN_Pa_ escape mutant of ΦM1 on UvrA mutant	This study
ΦM1-U7	ToxIN_Pa_ escape mutant of ΦM1 on UvrA mutant	This study
ΦM1-U8	ToxIN_Pa_ escape mutant of ΦM1 on UvrA mutant	This study
ΦM1-U9	ToxIN_Pa_ escape mutant of ΦM1 on UvrA mutant	This study
ΦM1-U10	ToxIN_Pa_ escape mutant of ΦM1 on UvrA mutant	This study
ΦM1-PL2	TenpIN_Pl_ escape mutant of ΦM1	This study

### ΦM1 genomic sequencing.

Bacteriophage DNA was extracted with phenol-chloroform, using Phase Lock Gel tubes (Eppendorf) and in accordance with the manufacturer's instructions, as for bacteriophage λ. The extracted DNA was subjected to pyrosequencing on a Roche 454 Genome Sequencer FLX at the DNA sequencing facility, Department of Biochemistry, University of Cambridge. Contiguous read segments (contigs) were assembled using Newbler (Roche). The ΦM1 wild-type sequence was determined in one lane of the sequencing run. The three escape phage genomes were individually tagged with independent identifying sequences and then combined and sequenced as a mixture within a second lane. For each of the four phages, the final assembled sequence consisted of a single contig of approximately 43,500 bp. The average read length was 250 bp. The wild-type sequence was assembled from 13,628 reads, leading to approximately 78× coverage of the full sequence. Escape phage ΦM1-A, -B, and -D sequences were assembled from 4,925, 5,188, and 5,886 reads, respectively, resulting in approximately 29× coverage of each sequence.

When the sequence data are viewed, beginning at bp 43572 (in the final ΦM1 wt sequence), there are 15 tandem repeats of the 2-bp sequence TG. The number of TG repeats varied between the raw sequences of each phage, from 17 in ΦM1-A to 1 in ΦM1-B and 7 in ΦM1-D. The exact number of TG repeats in each phage genome could not be accurately confirmed by sequencing a specific amplicon. Therefore, in order to sequence this region, it was specifically amplified (primers TRB107/TRB108 and TRB115/TRB116) and cloned into pBR322 (NEB). From the resulting plasmid DNA, the region was successfully sequenced on both forward and reverse strands.

Potential ORFs were identified using gene prediction tools such as ORFfinder (https://www.ncbi.nlm.nih.gov/orffinder/), GeneMark.hmm (47), and Glimmer (48), along with BLAST (31) homology searches and manual annotation. RBSfinder (49) was used to predict ribosome-binding sites (see Table S1 in the supplemental material). ΦM1 tRNAs were identified using tRNAScan-SE (50). The BDGP Neural Network Promoter Prediction (51) program did not identify any consensus promoters. The program Stretcher, from the EMBOSS suite (http://www.ebi.ac.uk/Tools/psa/emboss_stretcher/nucleotide.html), was used for global nucleotide alignments. The ΦM1 genome was viewed and annotated using Artemis (52).

### Plasmid construction.

Molecular biology techniques were performed as described previously (53). All primers were obtained from Sigma-Genosys and Invitrogen and are listed in Table 4. All plasmids constructed and/or used in this study are listed in Table 5, along with the primers used for their construction. All recombinant plasmid sequences were verified by DNA sequencing.

**TABLE 4:** Primers used in this study

Primer	Sequence (5′–3′)	Description	Restriction site
KDOI	TTTTGGATCCGTTTTATCGACATTGTGAACC	*toxIN* locus	BamHI
PF147	GTATCTAGAGTAGTCGCCTCTTTTACTTTATTAC	*toxI*	XbaI
PF217	TTGTATACTTAAGTTATTGACTCTATAGCTCAG	ToxI amplification for S1 nuclease protection assay	HindIII
PF218	TTGACTATGTAGTCGCCTCTTTTACTTTATTTCGAACCTCGGACCTGCG	ToxI amplification for S1 nuclease protection assay	DrdI
TRB37	CCGGCATATGAAATTCTACACTATATCAAGC	Used for ToxIN CBD	NdeI
TRB38	GTGGTTGCTCTTCCGCACTCGCCTTCTTCCGTAT	Used for ToxIN CBD	SapI
TRB107	TTGAATTCTGCGCAAGCAACTGGTGCACC	ΦM1 sequencing primer	EcoRI
TRB108	TTAAGCTTCTTGAATCTGTACTCACCG	ΦM1 sequencing primer	HindIII
TRB111	TTGAATTCCTGTAGGAGCGTGGAATGC	ΦM1 escape locus	EcoRI
TRB115	TTGAATTCCAGGGGTGTTACCTACTCC	ΦM1 sequencing primer	EcoRI
TRB116	TTAAGCTTGTAACTGTGCAGTGATACC	ΦM1 sequencing primer	HindIII
TRB117	TTGAATTCCCTACAATGCCCCAGATGC	ΦM1 escape locus	EcoRI
TRB118	TTAAGCTTACGGTCGTACTTGGCTTCG	ΦM1 escape locus	HindIII
TRB125	TTAAGCTTCTAATCCTACGCCTTGTGC	ΦM1 escape locus	HindIII
TRB126	TTGAATTCAAGGTGGATGCAACTCGGG	ΦM1 escape locus	EcoRI
TRB127	TTAAGCTTCTCTACATCATCCAACATC	ΦM1 escape locus	HindIII
TRB128	TTGAATTCGAGCTGCGTGATGAGTTCC	ΦM1 escape locus	EcoRI
TRB129	TTGAATTCGCTTACCCGATTATATCC	ΦM1 escape locus	EcoRI
TRB130	TTGAATTCCCAATTTAAAATTAATGA	ΦM1 escape locus	EcoRI
TRB134	TTAAGCTTATTACTTGTCATCGTCGTCCTTGTAGTCTCCTAGGTACCCCATCTGG	ΦM1 construct 7/ORF23 FLAG	HindIII
TRB135	TTAAGCTTAGTGATGGTGATGGTGATGTCCTCCTAGGTACCCCATCTGG	ΦM1 construct 7/ORF23-6His	HindIII
TRB332	TTAAGCTTATTACTTGTCATCGTCGTCCTTGTAGTCTCCCAGCATCGGCTTAAGGAAGCG	*uvrA*-FLAG	HindIII
TRB337	ATTAGGATCCGATAAGATCGAAGTTCG	*uvrA* primer	BamHI
TRB338	ATTAAAGCTTTTACAGCATCGGCTTAAG	*uvrA* primer	HindIII
UvrA dnF	TTTATTCCGGGAAGTGTGTGAATTTAAATTAGCGAGAGGCCAAATCATG	Fwd, 500 bp downstream of *uvrA*	SwaI
UvrA dnR	TTATCAGAATTCCTGCCGTGCAGGCAGTTCAG	Rev, 500 bp downstream of *uvrA*	EcoRI
UvrA upF	TTATCATCTAGATTGCAGTGCGCCTTCGATG	Fwd, 500 bp upstream of *uvrA*	XbaI
UvrA upR	CATGATTTGGCCTCTCGCTAATTTAAATTCACACACTTCCCGGAATAAA	Rev, 500 bp upstream of *uvrA*	SwaI

**TABLE 5:** Plasmids used in this study

Name	Description	Construction source or primers	Template	Resistance
pACYC184	Cloning vector	59		Cm
pBR322	E. coli cloning vector	NEB		Ap, Tc
pFR2	Photorhabdus luminescens TT01 full TenpIN_Pl_ locus	23	pBR322	Ap
pKNG-uvrA	UvrA marker exchange construct	UvrA upF, UvrA upR, UvrA dnF, UvrA dnR	pKNG101	Tc, Kan
pKNG101-Tc^r^	Marker exchange suicide vector	60		Tc
pMAT7	SdhE-FLAG expression vector	54	pBAD30	Ap
pMJ4	ToxI_Pa_, ToxN_Pa_-FLAG with native promoter in pBR322	29	pBR322	Ap
pQE80L	Protein expression vector	Qiagen		Ap
pRW50	Promoterless LacZ	38		Tc
pTA46	ToxIN_Pa_ with native promoter	29	pBR322	Ap
pTA104	ToxIN_Pa_ promoter	22	pRW50	Tc
pTA110	*In vitro* transcription vector for antisense ToxI_Pa_ RNA	PF217, PF218	pBSII SK^−^	Ap
pTRB18-KP14	ToxI_Pa_ containing	KDO1, PF147	pACYC184	Cm, Tc
pTRB14	ToxN_Pa_ CBD	TRB37, TRB38	pTA46	Ap
pTRB113	ΦM1 wt construct 3	TRB126, TRB118	pBAD30	Ap, glu
pTRB114	ΦM1 wt construct 4	TRB117, TRB127	pBAD30	Ap, glu
pTRB115	ΦM1 wt construct 5	TRB126, TRB125	pBAD30	Ap, glu
pTRB116	ΦM1 wt construct 6	TRB128, TRB118	pBAD30	Ap, glu
pTRB121	ΦM1-B construct 2	TRB117, TRB125	pBAD30	Ap, glu
pTRB123	ΦM1-B construct 4	TRB117, TRB127	pBAD30	Ap, glu
pTRB124	ΦM1-B construct 5	TRB126, TRB125	pBAD30	Ap, glù
pTRB133	ΦM1 wt construct 7	TRB111, TRB125	pBAD30	Ap, glu
pTRB134	ΦM1 wt construct 8	TRB129, TRB125	pBAD30	Ap, glu
pTRB135	ΦM1 wt construct 9	TRB130, TRB125	pBAD30	Ap, glu
pTRB136	ΦM1-A construct 7	TRB111, TRB125	pBAD30	Ap, glu
pTRB139	ΦM1-B construct 7	TRB111, TRB125	pBAD30	Ap, glu
pTRB140	ΦM1-B construct 8	TRB129, TRB125	pBAD30	Ap, glu
pTRB141	ΦM1-B construct 9	TRB130, TRB125	pBAD30	Ap, glu
pTRB148	ΦM1 wt construct 7-FLAG	TRB111, TRB134	pBAD30	Ap, glu
pTRB151	ΦM1-O construct 7-FLAG	TRB111, TRB134	pBAD30	Ap, glu
pTRB153	ΦM1-W construct 7-FLAG	TRB111, TRB134	pBAD30	Ap, glu
pTRB154	ΦM1-Y construct 7-FLAG	TRB111, TRB134	pBAD30	Ap, glu
pTRB155	ΦM1-D construct 7	TRB111, TRB125	pBAD30	Ap, glu
pTRB156	ΦM1-O construct 7	TRB111, TRB125	pBAD30	Ap, glu
pTRB157	ΦM1-V construct 7	TRB111, TRB125	pBAD30	Ap, glu
pTRB158	ΦM1-W construct 7	TRB111, TRB125	pBAD30	Ap, glu
pTRB159	ΦM1-Y construct 7	TRB111, TRB125	pBAD30	Ap, glu
pTRB160	ΦM1 wt LacZ fusion construct	TRB117, TRB127	pRW50	Tc
pTRB161	ΦM1 wt LacZ fusion construct	TRB111, TRB127	pRW50	Tc
pTRB162	ΦM1 wt LacZ fusion construct	TRB126, TRB127	pRW50	Tc
pTRB163	ΦM1-O LacZ fusion construct	TRB117, TRB125	pRW50	Tc
pTRB164	ΦM1 wt LacZ fusion construct	TRB117, TRB125	pRW50	Tc
pTRB189	ΦM1-23-6His	TRB111, TRB135	pQE-80L	Ap
pTRB190	ΦM1-O-23-6His	TRB111, TRB135	pQE-80L	Ap
pTRB300	UvrA-FLAG	TRB330, TRB332	pBAD33	Cm, glu
pTRB301	UvrA-6His	TRB337, TRB338	pQE-80L	Ap

### Measuring ToxI_Pa_ and ToxN_Pa_ levels during phage infection.

Two cultures of 180 ml of LB containing Ap were inoculated with 2-ml overnight cultures of P. atrosepticum(pBR322) or P. atrosepticum(pMJ4), respectively. Cultures were grown at 25°C and shaken at 180 rpm to an optical density at 600 nm (OD_600_) of 1, and each was split into two 80-ml volumes, one of which was infected with phage at a multiplicity of infection (MOI) of 1, while the other served as a negative control without infection. Cultures were left for 10 min without shaking for phage adsorption and then shaken at 25°C and 180 rpm. Samples for OD_600_ measurement, RNA preparation, and protein analysis were taken regularly during infection. Total RNA was isolated using the TRIzol method and subsequently DNase treated. Cell pellets for Western blot analysis were resuspended in 1× phosphate-buffered saline (PBS) according to the OD_600_ measurement.

### Western blot analysis of ToxN_Pa_ during infection.

One-milliliter samples of the cell cultures were taken, pelleted, and resuspended in 1× PBS according to the OD_600_. For samples taken during ΦM1 phage infection, the protein was quantified using a NanoDrop spectrophotometer (ThermoScientific), and equal amounts of protein (150 μg) were resolved by 12% PAGE. Proteins were transferred to a polyvinylidene difluoride (PVDF) membrane and blocked for 1 h in 1× PBS containing 5% milk powder. Immunodetection of FLAG-tagged ToxN was performed overnight at 4°C in 1× PBS using anti-FLAG M2 antibody (Sigma). Goat anti-mouse IgG-horseradish peroxidase (HRP) (Santa Cruz) was used as a secondary antibody. Bands were visualized on X-ray film using the SuperSignal West Pico chemiluminescent substrate kit (Pierce). SdhE-FLAG expressed from pMAT7 (54) was used as a control in the blot tracking ΦM1 infection.

### S1 nuclease protection assays.

An antisense probe covering the complete ToxI_Pa_ sequence was made by amplification of the ToxI_Pa_ locus from plasmid pTA110, using primers PF217 and PF218, and subsequent *in vitro* transcription and gel extraction of the probe as described previously (55), generating a uniformly [^32^P]UTP-labeled antisense transcript. Ten micrograms of DNase-treated total RNA was hybridized to the antisense probe overnight at 68°C in a total volume of 30 μl containing 22% or 6% formamide for the ΦM1 or ΦM1-O total RNA, respectively, 40 mM PIPES [piperazine-*N*,*N*′-bis(2-ethanesulfonic acid)]-KOH (pH 6.4), 1 mM EDTA, and 400 mM NaCl. Reaction mixtures were treated with S1 nuclease (Invitrogen) (1 U μl^−1^) for 1.5 h at 37°C in a total volume of 300 μl 1× S1 nuclease buffer to degrade any single-stranded nucleic acids. Double-stranded hybridization products were precipitated, resuspended, and resolved by 10% PAGE. Bands were visualized by phosphorimaging (Bio-Rad Personal FX phosphorimager).

### Toxicity assays.

When required, media were supplemented with Ap, d-glucose (glu) at 0.2% (wt/vol), and l-arabinose (L-ara) at 0.1% (wt/vol). P. atrosepticum strains containing two plasmids were grown as 10-ml overnight cultures, used to inoculate 25 ml LB, Ap, Cm, and glu in 250-ml conical flasks, and grown at 25°C and 250 rpm, from a starting OD_600_ of ∼0.04, until exponential phase (∼1 × 10^8^ CFU ml^−1^). Samples were removed, washed with PBS, serially diluted, and plated for viable counts at 25°C on LBA-Ap-Cm plates containing either (i) glu, to repress expression, or (ii) l-ara, to induce expression. Single-plasmid strains were treated in the same way, except that Cm was omitted from the growth conditions.

### β-Galactosidase assays.

Liquid assays for LacZ activity were performed using the substrate 4′-methylumbelliferyl-β-d-glucuronide (MUG) as described before (56). Briefly, samples of culture (150 μl) were taken at each time point and frozen at −80°C until required. Ten-microliter aliquots of each sample culture were frozen at −80°C for 10 min and then thawed at room temperature. Next, 100 μl reaction buffer (PBS, 400 μg ml^−1^ lysozyme, 250 μg ml^−1^ MUG) was added, and samples were immediately monitored in a Gemini XPS plate reader with the following parameters: excitation, 360 nm; emission, 450 nm; cutoff, 435 nm; eight reads per well; measurements taken every 30 s for 30 min. Relative fluorescence units min^−1^ was calculated from a period of linear increase in fluorescence, normalized to the OD_600_ of the sample.

### Pulldown of ToxIN_Pa_ and M1-23 from cell lysates.

Using ΦM1 and ΦM1-O genomic DNA, ΦM1-23 and M1-O-23 were amplified via PCR using TRB111 and TRB135 as primers. The products were then digested using the relevant restriction enzymes, ligated into pQE-80L, and then used to transform ER2566. For the ToxIN_Pa_ strains, pMJ4 (which contains ToxIN_Pa_-FLAG) was used, and a new plasmid was constructed to make a ToxN_Pa_-chitin binding domain (CBD) fusion. This was produced using pTA46 and primers TRB37 and TRB38. The plasmid pTRB14 was then used to transform ER2566, which had previously been transformed with pTRB18-KP14, which contains a ToxI_Pa_ sequence.

Expression strains were grown in 2× YT medium (per liter, 16 g tryptone, 10 g yeast extract, 5 g NaCl) at 37°C until an OD_600_ of approximately 1. The cultures were then induced with the appropriate supplement (0.5 mM IPTG for M1-23-6His and M1-O-23-6His) and then left to grow overnight at 18°C. No inducers were added to the tagged ToxIN_Pa_-containing strains, as ToxIN_Pa_ is constitutively expressed on pBR322.

Cells were harvested by centrifugation at 8,000 × *g*, and the pellets were resuspended in 10 ml lysis buffer (50 mM NaH_2_PO_4_·2H_2_O, 500 mM NaCl, 10 mM imidazole, 10% glycerol, pH 8.0) per 500 ml of original culture volume. Cells were then lysed by four passes through a high-pressure homogenizer (EmulsiFlex; at up to 15,000 lb/in^2^). Lysed cells were centrifuged at 8,000 × *g*, and the supernatants were kept for further coimmunoprecipitation experiments.

In the experiments using M1-23-6His and M1-O-23-6His as bait, 1.5 ml Ni^2+^ resin columns were used with ToxIN_Pa_-FLAG. The columns were equilibrated using 3 column volumes (CV) of lysis buffer before the His-tagged protein lysates were loaded onto the resin. Loaded resins were washed with 5 CV of wash buffer 1 (20 mM imidazole), followed by 10 CV of wash buffer 2 (40 mM imidazole). The FLAG-tagged ToxIN_Pa_ was then loaded onto the appropriate columns via continuous flow for at least 3 h (often overnight) before washing with 5 CV wash buffer 1 and 10 CV wash buffer 2.

Samples were eluted from the resin using elution buffer (250 mM imidazole) via three 1-ml fractions and analyzed by Western blot analysis using antibodies against His (Novagen) and FLAG (Sigma) tags. Briefly, samples were run on 12.5% Tris-Tricine gels and transferred onto Immobilon-P PVDF membranes (pore size, 0.45 μm; Millipore) at 250 mA for 90 min. Membranes were then blocked with a 5% milk plus PBST (PBS with Tween 20) solution for 1 h before incubation with anti-His and anti-FLAG antibodies at 1:10,000 for 2 h. After incubation, the membranes were washed three times for 5 min each in PBST and then incubated with the secondary anti-mouse antibody (Sigma) at 1:10,000 for 1 h before they were washed again three times for 5 min each in PBST. The blots were then probed with Immobilon-Western chemiluminescent HRP-substrate (Millipore) and developed.

For experiments in which ToxIN_Pa_ was used as the bait, the strain expressing ToxIN_Pa_-CBD was used with 1 ml chitin resin. The protocol and buffers used were as described by the manufacturer (NEB). Briefly, the ToxIN_Pa_-CBD lysate was loaded onto the column and washed with 40 ml of column buffer. The M1-23 or control pQE-80L lysates were then added to their respective columns. The columns were washed twice with 10 ml and then 27 ml of column buffer, followed by a dithiothreitol (DTT) flush, 5 to 7 ml for 10 min. Columns were then left to incubate overnight at room temperature. After incubation, elution was carried out using 15 ml of column buffer. Western blot analyses were then performed on the samples as previously described.

### Measuring ToxI_Pa_ levels after ToxIN_Pa_ pulldown with M1-23.

ToxI_Pa_ levels were measured in the eluted fractions of the ToxIN_Pa_-CBD chitin resin column experiments. Samples from cultures either expressing M1-23 or containing the pQE-80L vector control were separated by electrophoresis at 80 V, using a 1% (wt/vol) agarose gel made with 0.5× TAE (Tris-acetate-EDTA). Additionally, samples were also measured with a NanoDrop spectrophotometer (Labtech; ND-1000).

### Coimmunoprecipitation of UvrA and M1-23.

UvrA-6His was constructed by amplification from the E. coli W3110 genome using primers TRB337 and TRB338. PCR products were then digested with the appropriate restriction enzymes, and the digested product was purified and then ligated into pQE-80L to generate UvrA with an N-terminal His tag, pTRB301. This plasmid was then used to transform the E. coli expression strain ER2566. Likewise, UvrA-FLAG was constructed in a similar way but using primers TRB330 and TRB332 and ligated into pBAD33.

Expression and subsequent experiments were performed as described earlier using His-tagged proteins as bait on Ni^2+^ resin. Expression of UvrA-FLAG was induced by the addition of 0.02% arabinose.

### Construction of the P. atrosepticum *uvrA* mutant.

The *uvrA* mutant of P. atrosepticum was constructed via allelic exchange. This was performed using the plasmid pKNG-uvrA, which was derived from pKNG101. The plasmid was constructed by first amplifying 500-bp regions up- and downstream of the *uvrA* gene in P. atrosepticum SCRI1043. These two sequences were then ligated together with a kanamycin cassette inserted in between.

The suicide vector derivative pKNG-uvrA was used to transform E. coli β2163 and grown overnight in the appropriate selective medium. This served as the donor strain and, along with an overnight culture of the recipient strain, P. atrosepticum SCRI1043, was pelleted and resuspended in LB. Both cultures were then mixed at the ratios of 2:1, 1:1, and 1:2 up to a final volume of 100 μl. The resulting mixtures were then spotted on DAPA-containing plates and incubated at 25°C for 24 h. After mating, the patches were resuspended in 100 μl LB, serially diluted, and spread onto LBA plates containing tetracycline. These plates were incubated for 2 days at 25°C, and colonies that appeared were picked and grown in LB overnight. The subsequent overnight cultures were serially diluted, and 50-μl samples were plated onto LBA plates containing 10% (wt/vol) sucrose. Colonies were also patched onto LBA plates containing kanamycin, and the gene deletion was confirmed using colony PCR and DNA sequencing. The strain was confirmed phenotypically as UvrA negative by demonstrating a hypersensitivity to UV light (see Fig. S1 in the supplemental material).

### Accession number(s).

The genome of ΦM1 has been submitted to GenBank under the accession number JX290549.

## Supplementary Material

Supplemental material
