# Supplementary material for: Evolution of Pectobacterium Bacteriophage ΦM1 To Escape Two Bifunctional Type III Toxin-Antitoxin and Abortive Infection Systems through Mutations in a Single Viral Gene
Source: Appl Environ Microbiol. 2017 Mar 31;83(8):e03229-16. doi: 10.1128/AEM.03229-16 (PMC5377504; doi:10.1128/AEM.03229-16)
Supplement: Supplemental material [file supp_83_8_e03229-16__index.html]

Evolution of Pectobacterium Bacteriophage ΦM1 To Escape Two Bifunctional Type III Toxin-Antitoxin and Abortive Infection Systems through Mutations in a Single Viral Gene — Supplemental material 

# Evolution of Pectobacterium Bacteriophage ΦM1 To Escape Two Bifunctional Type III Toxin-Antitoxin and Abortive Infection Systems through Mutations in a Single Viral Gene

## Supplemental material

- Supplemental file 1 -

  UV sensitivity test against *P. atrosepticum* and the *uvrA* mutant (Fig. S1); restriction digest analysis of ΦM1 wild-type and escape phages (Fig. S2); *phiM1-23* has a weak promoter upstream (Fig. S3); details of ORFs and tRNA within the ΦM1 wild-type genome (Table S1); M1-23 mutations (Table S2).

  PDF, 1009K
