# Supplementary material for: Evolution of Pectobacterium Bacteriophage ΦM1 To Escape Two Bifunctional Type III Toxin-Antitoxin and Abortive Infection Systems through Mutations in a Single Viral Gene
Source: Appl Environ Microbiol. 2017 Mar 31;83(8):e03229-16. doi: 10.1128/AEM.03229-16 (PMC5377504; doi:10.1128/AEM.03229-16)
Supplement: Supplemental material [file AEM.03229-16_zam999117762s1.pdf]

Supplemental material

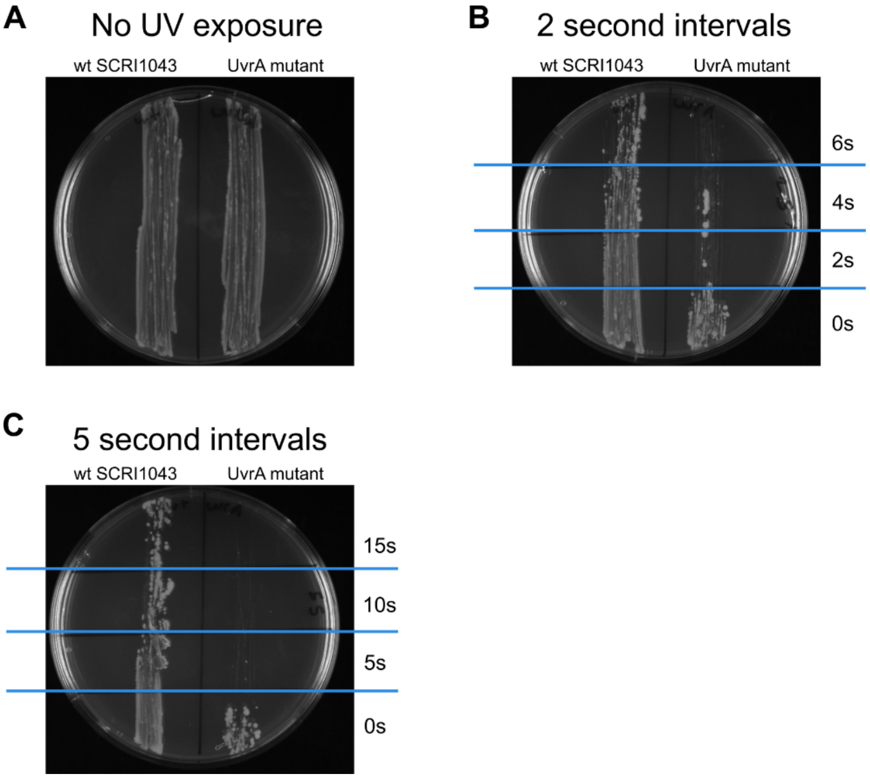

FIG S1 UV sensitivity test against *P. atrosepticum* and the *uvrA* mutant. (A) Without exposure to UV light there is no observable difference in growth between wt SCRI1043 and the *uvrA* mutant. (B), and (C) show different UV exposures at two and five seconds intervals respectively. The growth of the *uvrA* mutant is significantly reduced in these conditions while the wt strain shows a less dramatic effect.

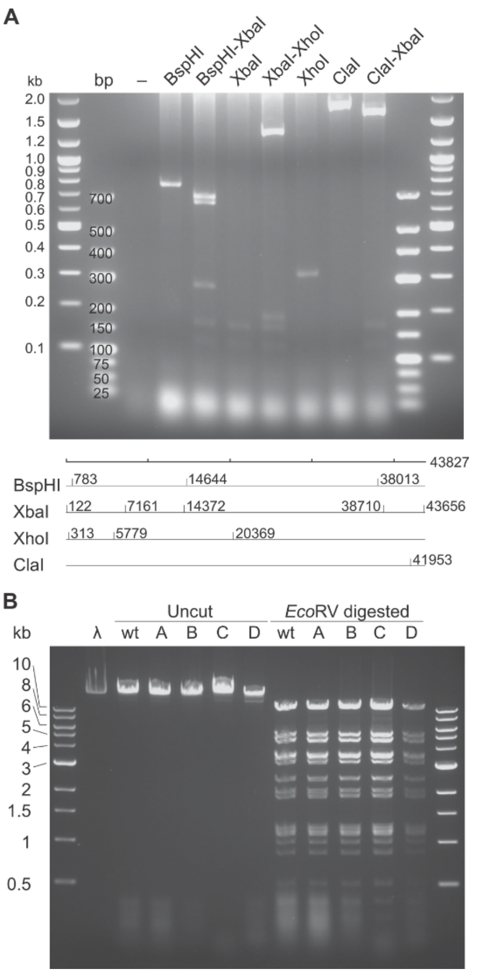

FIG S2 Restriction digest analysis of ΦM1 wt and escape phages. (A) ΦM1 wt contains DTRs of approximately 293 bp. Fourteen µg of ΦM1 wt genomic DNA was digested with the indicated enzymes for 3 h, then separated by gel electrophoresis within a 2% agarose gel. The restriction pattern matched the predicted digest from the finalised ΦM1 wt genomic sequence, as indicated below the gel image. An uncut sample lane is shown in "-". (B) There are no gross genetic changes between ΦM1 wt and the selected escape phages ΦM1 A, B, C and D. Genomic DNA from each phage was digested with *Eco*RV for 3 hours then separated by gel electrophoresis within a 1% agarose gel. Genomic DNA from phage λ (48,502 bp) was used as a high molecular weight marker for genomic DNA sizing.

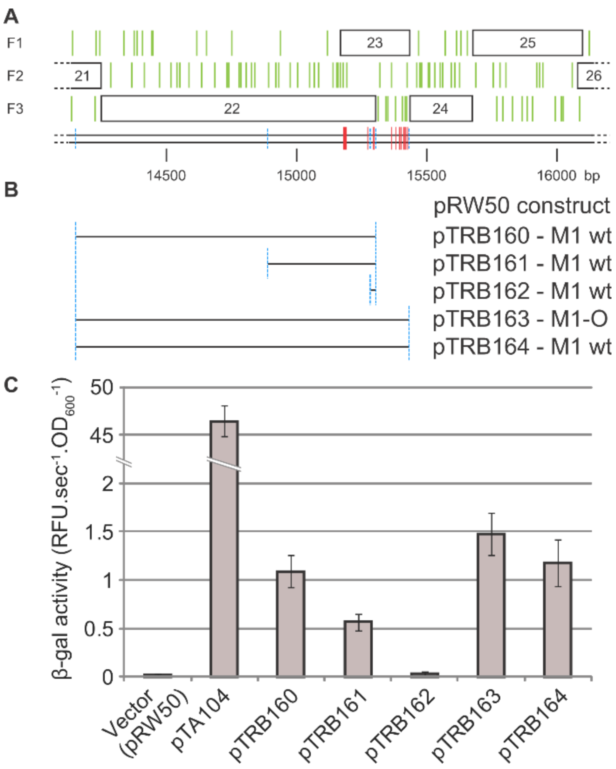

FIG S3 *phiM1-23* has a weak promoter upstream. (A) and (B) Sections of the ΦM1 escape locus were cloned into the LacZ reporter plasmid pRW50. Blue dotted lines in (A) reflect the construct boundaries in (B). The figure is drawn to scale. (C) These constructs were tested for LacZ activity in liquid assays. There is a promoter immediately upstream of *phiM1-23*, though the activity is weak compared to the positive control, pTA104, which contains the constitutive ToxIN$_{Pa}$ promoter.

TABLE S1 Details of ORFs and tRNA within the ΦM1 wild type genome

Gene	Start position (bp)	End position (bp)	Length (bp)	Size (aa)	E value	*Significant BLAST hit (Organism)	Accession No.
phiM1-01	785	940	156	52	1×10^{-7}	Hypothetical protein SMR0164 (*Serratia marcescens* plasmid R478)	NP_941234
phiM1-02	1348	1542	195	65	N/A	No significant homologue	-
phiM1-03	1542	1730	189	63	N/A	No significant homologue	-
phiM1-04	1868	2434	567	189	2×10^{-16}	Hypothetical protein, gp2 (*Vibrio* phage VP93)	ACP44073
phiM1-05	2501	2761	261	87	N/A	No significant homologue	-
phiM1-06	2841	2948	108	36	N/A	No significant homologue	-
phiM1-07	3105	3281	177	59	N/A	No significant homologue	-
phiM1-08	3281	3622	342	114	1×10^{-4}	Hypothetical protein, gp5 (*Vibrio* phage VP93)	ACP44076
phiM1-09	3643	5373	1731	577	N/A	No significant homologues	-
phiM1-10	5366	6334	969	323	4×10^{-31}	Putative peptidase, gp7 (*Vibrio* phage VP93)	ACP44078
phiM1-11	6324	6755	432	144	N/A	No significant homolog	-
phiM1-12	6888	7730	843	281	$4 \times 10\text{-}^{27}$	Putative primase DnaG, gp10 (*Vibrio* phage VP93)	ACP44081
phiM1-13	7821	8054	234	78	N/A	No significant homolog	-
phiM1-14	8058	9317	1260	420	2×10^{-117}	DnaB, putative DNA helicase, gp23 (*Pseudomoas* phage LKA1)	CAK24991
phiM1-15	9586	9792	207	69	N/A	No significant homolog	-
phiM1-16	9827	10051	225	75	N/A	No significant homolog	-
phiM1-17	10047	10343	297	99	N/A	No significant homolog	-
phiM1-18	10339	10518	180	60	N/A	No significant homolog	-
phiM1-19	10588	11070	483	161	2×10^{-23}	Hypothetical protein, Orf78 (*Pseudomonas* phage D3)	AAF80834
phiM1-20	11045	13384	2340	780	0.0	Putative DNA polymerase, gp14 (*Vibro* phage VP93)	ACP44085
phiM1-21	13406	14239	834	278	2×10^{-31}	Hypothetical protein, gp31 (*Pseudomonas* phage LKA1)	CAK24999
phiM1-22	14256	15299	1044	348	1×10^{-69}	Putative DNA exonuclease, gp32 (*Pseudomonas* phage LKA1)	CAK25000
phiM1-23	15169	15423	255	85	N/A	No significant homolog	-
phiM1-24	15423	15665	243	81	N/A	No significant homolog	-
phiM1-25	15661	16083	423	141	8×10^{-34}	Putative DNA endonuclease, gp33 (*Pseudomonas* phage LKA1)	CAK25001
phiM1-26	16067	16285	219	73	N/A	No significant homolog	
phiM1-27	16450	17421	972	324	3×10^{-72}	Hypothetical phosphatase, gp23 (*Enterobacteria* phage SP6)	AAP48762
phiM1-28	17535	17900	366	122	8×10^{-26}	PseT polynucleotide kinase, gp27 (*Erwinia amylovora* phage Era 103)	ABM63417
phiM1-29	17900	18199	300	100	1×10^{-13}	Hypothetical protein, gp35 (*Pseudomonas* phage LKA1)	CAK25003
phiM1-30	18317	18496	180	60	N/A	No significant homolog	-
phiM1-31	18506	20947	2442	814	9×10^{-101}	T3/T7-like RNA polymerase, gp37 (*Pseudomonas* phage LKA1)	CAK25005
phiM1-32	21043	21237	195	65	N/A	No significant homolog	-

*BLAST searches performed 2012

TABLE S1 continued. Details of ORFs and tRNA within the ΦM1 wild type genome

Gene	Start position (bp)	End position (bp)	Length (bp)	Size (aa)	E value	*Significant BLAST hit (Organism)	Accession No.
phiM1-33	21215	21625	411	137	N/A	No significant homolog	-
phiM1-34	21645	21944	300	100	N/A	No significant homolog	-
phiM1-35	21950	23458	1509	503	$4\ x10^{-120}$	Putative head-tail connector protein, gp27 (*Vibrio* phage VP93)	ACP44098
phiM1-36	23461	24327	867	289	$2\ x10^{-15}$	Putative scaffolding protein, gp28 (*Vibrio* phage VP93)	ACP44099
phiM1-37	24344	24799	456	152	$1\ x10^{-7}$	Putative HNH endonuclease, gp21 (*Enterobacteria* phage T7)	AAP33928
phiM1-38	24799	25779	981	327	$8\ x10^{-82}$	Putative capsid protein, gp29 (*Vibrio* phage VP93)	ACP44100
$tRNA^{Ile}$	25840	25937	98	N/A		Detected de novo using tRNAScan	-
phiM1-39	259555	26509	555	185	$3\ x10^{-29}$	Putative tail tubular protein A, gp30 (*Vibrio* phage VP93)	ACP44101
phiM1-40	26522	28849	2328	776	$2\ x10^{-145}$	Putative tail tubular protein B, gp31 (*Vibrio* phage VP93)	ACP44102
phiM1-41	28849	29460	612	204	$7\ x10^{-6}$	Hypothetical protein, gp32 (*Vibrio* phage VP93)	ACP44103
phiM1-42	29473	32184	2712	904	$5\ x10^{-9}$	Putative injection needle component, gp33 (*Vibrio* phage VP93)	ACP44104
phiM1-43	32197	35985	3789	1263	$1\ x10^{-80}$	Putative internal core protein, gp34 (*Vibrio* phage VP93)	ACP44105
phiM1-44	35998	37599	1602	534	$2\ x10^{-22}$	Putative tail-fibre protein, gp35 (*Vibrio* phage VP93)	ACP44106
phiM1-45	37611	37895	285	95	$1\ x10^{-12}$	Putative DNA maturase A, gp37 (*Vibrio* phage VP93)	ACP44108
phiM1-46	37907	39721	1815	605	0.0	Putative DNA maturase B, gp38 (*Vibrio* phage VP93)	ACP44109
phiM1-47	39733	39918	186	62	N/A	No significant homolog	
phiM1-48	39932	40468	537	179	$5\ x10^{-5}$	Putative structural protein, gp56 (*Pseudomonas* phage LKA1)	CAK25024
phiM1-49	40481	40864	384	128	$2\ x10^{-10}$	Hypothetical protein, gp65 (*Enterobacteria* phage RTP)	CAJ42269
phiM1-50	40860	41123	264	88	2.8	Hol, putative holing protein, Orf28 (*Pasteurelia* phage F108)	AAZ93663
phiM1-51	41110	41712	603	201	$1\ x10^{-9}$	Putative lysozyme-like endolysin, gp37 (*Erwinia* phage ΦEa21-4)	ACH88950
phiM1-52	41730	43487	1758	586	$3\ x10^{-36}$	Tailspike protein (*Salmonella* phage Det7)	CA078738

*BLAST searches performed 2012

TABLE S2 M1-23 mutations

Phages	Mutation relative to ΦM1 wt	Location relative to *phiM1-23*	Effect on M1-23	Effect on exonuclease	Number of phages
ΦM1-E1<br>ΦM1-E2<br>ΦM1-E3<br>ΦM1-E4	15169 A to C	A1C	Met to Leu	Tyr to Ser	4
ΦM1-C<br>ΦM1-E5<br>ΦM1-E6<br>ΦM1-E7<br>ΦM1-E8<br>ΦM1-E9<br>ΦM1-U4<br>ΦM1-U5<br>ΦM1-U6<br>ΦM1-U9	15170 T to C	T2C	Met to Thr	Tyr to Tyr	10
ΦM1-E10	15171 G to A	G3A	Met to Ile	Asp to Asn	1
ΦM1-E11	15175 A to T	A7T	Lys to Stop	Gln to Leu	1
ΦM1-E12	15256 A to T	A88T	Lys to Stop	Glu to Val	1
ΦM1-X<br>ΦM1-E13<br>ΦM1-E14<br>ΦM1-E15	15288 AA to A	AA120A	FS to Stop after 9aa	FS to Stop after 45aa	4
ΦM1-B<br>ΦM1-E16<br>ΦM1-E17<br>ΦM1-E18<br>ΦM1-E19<br>ΦM1-E20	15292 C to T	C124T	Arg to Stop	Pro to Leu	6
ΦM1-E21	15359 T to C	T191C	Leu to Pro	-	1
ΦM1-E22<br>ΦM1-E23	15384 T to G	T216G	Tyr to Stop	-	2
ΦM1-Y	15397 G to A	G229A	Asp to Asn	-	1
ΦM1-W	15398 A to T	A230T	Asp to Val	-	1
ΦM1-E24<br>ΦM1-E25	15398 A to C	A230C	Asp to Ala	-	2
ΦM1-U7	15398 A to G	A230G	Asp to Gly	-	1
ΦM1-O<br>ΦM1-Q<br>ΦM1-E26<br>ΦM1-E27	15407 A to C	A239C	Gln to Pro	-	4
ΦM1-D<br>ΦM1-E	15410 T to C	T242C	Met to Thr	-	2
ΦM1-E28	15410 T to G	T242G	Met to Arg	-	1
ΦM1-V<br>ΦM1-E29	15415 T to G	T247G	Tyr to Asp	-	2
ΦM1-A<br>ΦM1-E30	15416 A to C	A248C	Tyr to Ser	-	2

TABLE S2 continued. M1-23 mutations

Phages	Mutation relative to ΦM1 wt	Location relative to *phiM1-23*	Effect on M123	Effect on exonuclease	Number of phages
ΦM1-Z<br>ΦM1-E31<br>ΦM1-E32<br>ΦM1-E33<br>ΦM1-E34<br>ΦM1-E35<br>ΦM1-E36<br>ΦM1-E37<br>ΦM1-E38<br>ΦM1-E39<br>ΦM1-E40<br>ΦM1-E41<br>ΦM1-E42<br>ΦM1-E43<br>ΦM1-E44<br>ΦM1-E45<br>ΦM1-E46<br>ΦM1-U1<br>ΦM1-U2<br>ΦM1-U8<br>ΦM1-U10	15416 A to G	A248G	Tyr to Cys	-	21
ΦM1-E47	15419 T to C	T251C	Leu to Pro	-	1
ΦM1-E48<br>ΦM1-E49	15424 T to C	T256C	Stop to Arg<br>(extension of 10 aa)	-	2

Different mutations: 21 Total mutations: 70
